# In-silico analysis of cattle blood transcriptome to identify lncRNAs and their role during bovine tuberculosis

**DOI:** 10.1038/s41598-024-67001-0

**Published:** 2024-07-17

**Authors:** Priyanka Garg, Venkata Krishna Vanamamalai, Shailesh Sharma

**Affiliations:** 1 National Institute of Animal Biotechnology (NIAB), Opp. Journalist Colony, Near Gowlidoddi, Extended Q City Road, Gachibowli, Hyderabad, Telangana 500032 India; 2 Regional Centre for Biotechnology, Faridabad-Gurgaon Expressway, Faridabad Rd, Faridabad, Haryana 121001 India

**Keywords:** Long noncoding RNA, Bovine tuberculosis (BTB), In-silico transcriptome analysis, Differential expressed analysis, Functional annotation, Co-expression analysis, Public data analysis, RNA sequencing, Immunogenetics, Bacteriology, Gene ontology, Sequence annotation, Computational biology and bioinformatics

## Abstract

Long noncoding RNAs (lncRNAs) are RNA molecules with a length greater than 200 nucleotides that do not code for functional proteins. Although, genes play a vital role in immune response against a disease, it is less known that lncRNAs also contribute through gene regulation. Bovine tuberculosis is a significant zoonotic disease caused by *Mycobacterium bovis* (*M. bovis*) in cattle. Here, we report the in-silico analysis of the publicly available transcriptomic data of calves infected with *M. bovis*. A total of 51,812 lncRNAs were extracted across all the samples. A total of 216 genes and 260 lncRNAs were found to be differentially expressed across all the 4 conditions—infected vs uninfected at 8- and 20-week post-infection (WPI), 8 vs 20-WPI of both infected and uninfected. Gene Ontology and Functional annotation showed that 8 DEGs were annotated with immune system GOs and 2 DEGs with REACTOME immune system pathways. Co-expression analysis of DElncRNAs with DEGs revealed the involvement of lncRNAs with the genes annotated with Immune related GOs and pathways. Overall, our study sheds light on the dynamic transcriptomic changes in response to *M. bovis* infection, particularly highlighting the involvement of lncRNAs with immune-related genes. The identified immune pathways and gene–lncRNA interactions offer valuable insights for further research in understanding host–pathogen interactions and potential avenues for genetic improvement strategies in cattle.

## Introduction

One of the most significant zoonotic infectious diseases, Bovine tuberculosis (BTB) is caused by *Mycobacterium bovis* (*M. bovis*)^1^. BTB severely threatens livestock, human health, and economic well-being^2^. *M. tuberculosis* is the primary cause of tuberculosis (TB) in humans, while *M. bovis* is responsible for the disease mainly in animals. TB caused by both pathogens can be transmitted from animals to humans and vice versa^3^.

The respiratory system is the primary site of infection of *M. bovis*^4^. The primary routes of *M. bovis* transmission involve inhaling aerosols containing the bacteria, particularly in settings where infected animals, such as cattle, release respiratory secretions. Additionally, infection can occur through the consumption of raw milk obtained from diseased animals^5^. Consequently, bovine TB is primarily a respiratory infection, and most infections are believed to be spread between animals nearby through "direct" aerosol transmission^6^.

Analysing the transcriptome can offer valuable insights into complex diseases, shedding light on the genes responsible for immune responses^7^. Genes play a crucial role in determining the host immune response, but it's essential to recognize that long noncoding RNAs (lncRNAs) also contribute significantly by influencing gene expression through various mechanisms. These include participation in chromatin remodelling processes, acting as coactivators or corepressors with transcription factors, inhibiting translation, modulating splicing events, and influencing mRNA degradation by interacting with microRNAs. Importantly, despite not encoding proteins themselves, lncRNAs play a pivotal role in shaping cellular processes, exerting regulatory effects at both the RNA and protein levels^8,9^.

Previous studies have analysed transcriptome data of cows during BTB and revealed the role of different genes during BTB. They have observed the transcriptional upregulation of immune related genes during both the early and late phases of tuberculosis. These findings help for understanding the immune response during the disease^4^. Peripheral blood transcriptome analysis has revealed that the immune response in peripheral blood was similar to that observed at the primary site of BTB^1^. A study on whole blood transcriptome of cows infected with BTB has revealed the role of genes and their involvement in immune related pathways. But the role of lncRNAs was not reported in this study^7^. Previously, we have identified the role of genes and lncRNA in the host response in non-bovine hosts^10–12^. Another study has revealed the role of immune related genes and co-expressing lncRNAs during BTB in Primary Bovine Macrophages^13^. But such studies for identifying role of lncRNAs were not performed on the blood transcriptome of cows during BTB.

The primary objective of this study is to analyse the transcriptomic data obtained from whole blood cells of calves experimentally infected with *M. bovis* using the same pipeline, FHSpipe, as used in our previous study^11^. The focus is on identifying lncRNAs and comprehending the distinctive expression patterns of both lncRNAs and genes. The overarching goal is to deduce the role of lncRNAs during the immune response of cattle against bovine tuberculosis disease through co-expression analysis with differentially expressed genes.

## Materials and methods

### Transcriptomic data collection

The publicly available whole blood transcriptome data, comprising of 48 datasets, was obtained from the EBI-ENA database under the accession number PRJNA791899. This dataset was originally submitted by Abdelaal et al. from the University of Wisconsin-Madison^4^. Initially, they have taken a total of 12 Holstein calves, approx. 9 months of age and castrated males, of which, six were infected with a single dose of the virulent *M. bovis* strain 10-7428, while the remaining six calves were uninfected controls. Subsequently, whole blood samples for RNA isolation were procured from Holstein calves at two distinct time points: 8 weeks and 20 weeks post-infection (WPI) and sequenced using Illumina HiSeq 2000. These 48 RNA-Seq data samples were of whole blood from infected and uninfected control group (12 biological replicates per group) at 8- and 20-WPI of Holstein calves. The details of the samples are mentioned in Supplementary Table 1.

### Computational pipeline for analysis

The computational pipeline—FHSpipe, as described in a previous work on chicken^11^, was used. This pipeline includes quality filtering, mapping, assembly, identification of lncRNAs and generation of read counts for differential expression analysis. Further details regarding each of these steps are explained below.

### Quality filtering

Throughout the analysis pipeline, rigorous quality control measures were implemented to ensure data integrity and reliability. The downloaded samples underwent quality assessment, and a single tool, Fastp v 0.23.1^14^, was employed to filter out low-quality data and eliminate Illumina adaptor sequences. In standalone command line mode, Fastp was used with a read quality cut-off of 25. The analysis included the identification of overrepresented sequences, and comprehensive quality reports were generated in both HTML and JSON formats. The reads that underwent the filtering process were then primed for further downstream analysis.

### Mapping and assembly

The filtered reads underwent alignment to the cow reference genome using Hisat2 v 2.2.1^15^. The reference genome utilized for this alignment was the most recent version, ARS-UCD1.3. To annotate the transcripts, the Genome Feature Format (GFF) file corresponding to the latest genome annotation was utilized. The aligned reads were sorted and converted into Binary Alignment Map (BAM) format using Samtools v 1.10^16^. The assembled transcripts, derived from the Binary Alignment Files, were generated using Stringtie^17^ and were obtained in GFF format.

### Identification of long noncoding RNAs (lncRNAs)

GFFCompare v 0.12.6 was used to consolidate and annotate the transcripts from all samples into 16 different class codes^18^. Each class code represents a specific type of transcript. Sequences corresponding to class 'I' (intronic transcripts), class 'U' (unknown and intergenic transcripts), and class 'X' (antisense transcripts) were isolated using Bedtools v 2.27.1^19^. Subsequently, sequences with a length less than 200 nucleotides were filtered out. The remaining sequences underwent Open Reading Frame (ORF) prediction using ORFPredictor^20^. Transcripts with an ORF length of less than 100 amino acids were discarded. To assess functional domains, the retained sequences underwent a similarity search against the Pfam database^21^ using RPSBLAST from NCBI BLAST toolkit v 2.12.0^22^. Sequences with no known hits were further analysed for coding potential using CPC2 v 1.0.1^23^. Sequences with a noncoding tag were considered potential long noncoding RNAs. From the merged GTF file obtained through GFFCompare, a GFF file was extracted, containing details of transcripts identified by TCONS IDs associated with the extracted lncRNAs. This file was utilized in subsequent analyses, functioning as an annotation file for reassembly purposes. To enhance the reliability of the dataset, sequences were subjected to a similarity search against various databases, including tRNAdb^24^ and miRBase^25^, to eliminate any matching transcripts. Additionally, a search against NONCODE v6^26^ was conducted to identify both novel and known lncRNAs.

### Differential expression analysis

In accordance with the established pipeline, transcripts derived from Stringtie^17^ were employed to generate a merged GTF file utilizing the Merge function within Stringtie. This file encompasses transcripts assembled from all samples. In accordance with our prior studies^10,11^, the transcripts assembly was iteratively conducted, utilizing the merged GTF file as an annotation in Stringtie. This approach was adopted to enhance the precision of transcript quantification. Subsequently, read counts were extracted, and differential expression analysis was carried out using edgeR^27^ with the Generalised Linear Model (GLM). Differential expression analysis was performed under two conditions—infection-based analysis (infected v/s uninfected) and timepoint-based analysis (between timepoints 8 v/s 20 WPI). The differentially expressed genes (DEGs) and lncRNAs (DElncRNAs) with FDR corrected p-values < 0.05 were considered as significant DEGs and DElncRNAs. The visual representation of the chromosomal localization of the identified differentially expressed genes and lncRNAs was created using Circos v 0.69-9^28^.

### Gene ontology analysis

The standalone tool OmicsBox v2.1.14^29^ was utilized to perform gene ontology analysis and functional annotation of the differentially expressed genes (DEGs). Initially, sequences of DEGs were extracted using Bedtools 2.27.1^19^ and the reference genome. The extracted sequences were then loaded into Omicsbox for a blast search^30^ against the v5 non-redundant protein database, with cow (*Bos taurus*) (Tax ID 9913) as a taxonomy filter with an e-value of 1e-5. Following the blast search, EMBL-EBI Interpro scan^31^ was conducted against various databases, including protein families, domains, repeats, and sites. Subsequently, gene ontology mapping^32^ was performed using the latest GOA version 2022.03, followed by GO annotation. For functional annotation, EggNOG-Mapper v 2.1.0^33^ was utilized against EggNOG v 5.0.2. Combined pathway analysis was carried out against KEGG^34^ and Reactome^35^ databases. Visual representation of the results included illustrating gene ontologies of Biological Process, Molecular Function, and Cellular Component as GO level 2 pie charts. Additionally, the categories of Reactome and KEGG pathways were presented as bar plots. This comprehensive analysis provided insights into the functional characteristics and pathways associated with the identified differentially expressed genes.

### Co-expression analysis

The co-expression analysis of DEGs and DElncRNAs was performed using the R-based tool Weighted Gene Correlation Network Analysis (WGCNA) v1.71^36^. The extraction of expression values (FPKM) for all differentially expressed genes and lncRNAs from all samples utilized in-house Python scripts. These expression values were then employed as input for WGCNA. Upon loading the CSV file containing expression values, a thorough check for missing values in both samples and genes was conducted. Outlier samples were identified and subsequently removed. The selection of soft power involved choosing a scale-free topology fit index cut-off of 0.8. Based on the selected soft power, network adjacency, topology overlap matrix, and cluster dendrogram were generated. The minimum module size was determined from the dendrogram. Module eigengenes were computed, and similar modules were merged. The data of edges and nodes from the co-expression network were saved as TXT files. This process allowed for the identification of co-expression modules and exploration of relationships between genes and lncRNAs based on their expression patterns across samples. The generated network data can be further analysed for functional insights and regulatory relationships.

### Cis–trans analysis and lncRNA functional analysis

Utilizing the chromosomal localization data, an analysis was conducted to distinguish between "cis" and "trans" interactions for the identified co-expressing gene-lncRNA pairs. This categorization was based on the relative positions of the lncRNA and the co-expressing gene. "cis" interactions indicated the lncRNA's adjacency to the co-expressing gene on the same chromosome, while "trans" interactions signified the lncRNA and the co-expressing gene being located at different loci on the chromosome. This classification allowed for distinguishing between local (cis) and distant (trans) regulatory relationships between lncRNAs and their co-expressing genes.

Additionally, the functions of the lncRNAs were predicted indirectly based on the type of interaction, associating them with the known functions of the co-expressing genes. This approach provides insights into potential functional roles of the lncRNAs by leveraging the functions of genes they co-express with, as reported in relevant literature^37,38^.

### Gene–transcription factor interaction analysis

The MEME tool kit v5.4.1^39^ was employed to identify transcription factors that interact with the differentially expressed genes. Initially, the 5′ upstream sequences were extracted using Bedtools 2.27.1^19^. Subsequently, motifs in the 5′ UTR were identified using the MEME tool, with specific parameters such as –nod anr, -minw 8, -maxw 15, -maxsize 10,000,000,000, and –nmotifs 10. Following motif identification, the TomTom tool^39^ was utilized to determine transcription factors interacting with the identified motifs, employing the JASPAR2022 vertebrate database. The identified transcription factors were then cross-referenced with cow data obtained from the Animal Transcription Factor Database v3.0^40^. This approach allowed for the identification of potential transcription factors interacting with the differentially expressed genes, providing insights into the regulatory network associated with the observed gene expression changes.

### Gene-miRNA analysis

To identify microRNAs interacting with the differentially expressed genes, miRNet^41^, an online microRNA-centric network visual analysis platform, was employed. This platform integrates miRNA target data from four well-annotated databases: miRTarBase, TarBase, miRecords, and miRanda. The official gene symbols of all the Differentially Expressed Genes (DEGs) served as input for the analysis. The miRNet was employed to analyse the potential miRNAs interacting with the provided gene list. A list of potential miRNAs interacting with the DEGs was obtained from miRNet. The miRNA data, containing the identified interactions, was downloaded in CSV file format. This approach allowed for the exploration of microRNA-gene interactions, providing valuable information about potential regulatory relationships associated with the observed differential expression of genes.

### Network visualisation

The cis interactions between lncRNAs and genes, annotated with immune-related pathways and gene ontology, were extracted. Additionally, transcription factors interacting with the selected genes were also extracted and visualized using Cytoscape v3.9.1^42^ in the form of networks. This visualization provides a comprehensive view of the regulatory landscape involving lncRNAs, immune-related genes, and transcription factors, offering insights into potential regulatory mechanisms within the context of immune-related pathways and gene ontology.

## Results

### Quality filtering

The data of 48 samples was downloaded from the EBI-ENA database. The 48 samples are of 12 Holstein calves at 8- and 20-WPI. The quality control by Fastp showed that, on average, all 48 samples were passed with a value of 99.28%. Approximately 0.38% of reads were identified as low quality, while 0.31% were deemed short (refer to Supplementary Table 2). The average GC content was determined to be 46.84%, and following the filtering process, the Q30 base content reached 97.18%.

### Mapping and assembly

The pre-processed reads underwent mapping against the cow genome version ARS-UCD1.2, obtained from NCBI. The average mapping percentage across all samples was determined to be 98.17%, falling within a range of 97.83% to 98.37%. Subsequently, the mapped reads from all samples were assembled, revealing an average of 54,481 transcripts, with individual samples ranging from 53,883 to 55,120. After merging all the transcripts from all samples and reassembling the samples using Stringtie, the number of transcripts per sample was refined to 38,050. Detailed mapping and assembly results can be found in Supplementary Table 3. This process aims to ensure comprehensive coverage and accuracy in capturing the transcriptional landscape, facilitating downstream analyses and interpretations.

### Identification of long noncoding RNAs

**Figure 1:**
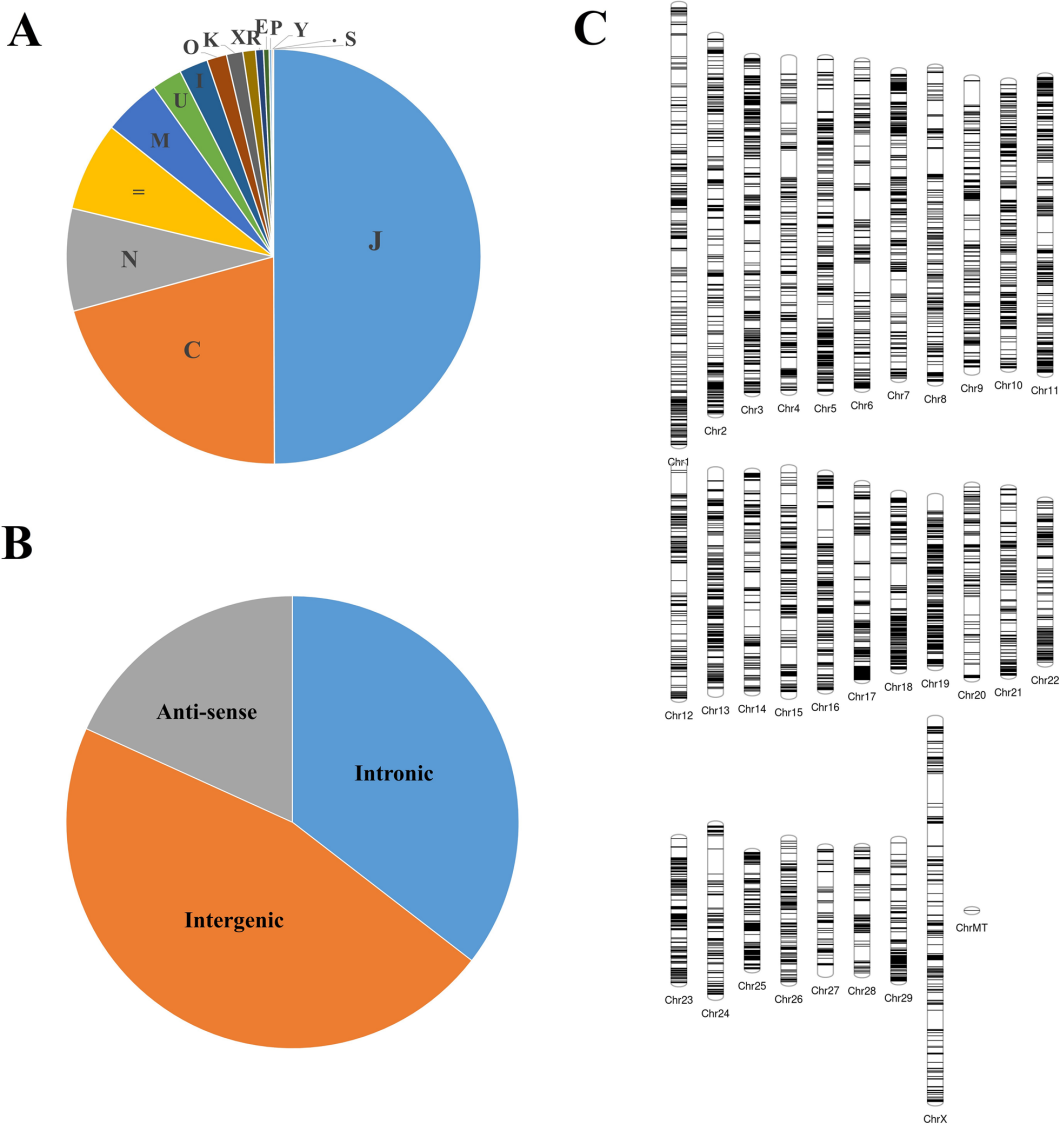
Identification of lncRNAs—(**A**) Pie chart showing the proportions of sequences of each class code. (**B**) Pie chart showing the proportions of each type of lncRNAs. (**C**) Figure showing the distribution of identified long noncoding RNAs across the chromosomes of cow.

After merging, a total of approximately 420,938 transcripts were acquired and annotated with 16 distinct class codes. As illustrated in Fig. 1A, nearly half (49.93%) of the transcripts were assigned Class code "J", denoting multi-exon transcripts with at least one junction match. Furthermore, 20.82% of the transcripts bore Class code "C", indicating transcripts contained in the reference (intron compatible). The remaining class codes were linked to less than 10% of the transcripts each. Table 1 provides further details, indicating that approximately 74,162 transcripts of class codes I, U, and X were extracted.

**Table 1:** Table showing the number of sequences eliminated and retained in each step during the extraction of long noncoding RNAs.

Step	Number of sequences eliminated	Number of sequences retained
Initial input	74,162	
Length filter	13,808	60,354
ORF filter	2646	57,708
Pfam filter	3156	54,552
CPC2	2740	51,812
Final	22,350	51,812

Following analysis using length filter, ORF filter, Pfam RPSBLAST, and CPC2 annotation, 51,812 transcripts successfully passed all filters, being considered potential lncRNAs. As depicted in Fig. 1B, among these, 18,373 transcripts were of class code I, 24,001 transcripts were of class code U, and 9,438 transcripts were of class code X. BLAST analysis against the NONCODE database v6 identified similarities in 21,708 out of 51,812 sequences. Among these, around 489 sequences showed both 100% similarity and coverage. It's noteworthy that there were no hits with 100% similarity in the miRBase (hairpin and mature) and tRNA databases. The chromosomal localization of the identified lncRNAs, plotted using the phenogram tool^43^, is depicted in Fig. 1C. This figure illustrates that chromosome 10 contained 6.77% of the identified lncRNAs. On average, chromosomes 5, 18, and 19 harboured approximately 5.69% of the lncRNAs, while chromosomes 27 and MT had the fewest number of identified lncRNAs.

### Differential expression analysis

Differential expression analyses were conducted for both genes and lncRNAs under two conditions: (I) between infected and uninfected states and (II) between different time points. Statistically, the genes and lncRNAs showing an FDR value < 0.05 were considered as significant. In infection-based analysis, about 6 genes and 87 lncRNAs were identified to be differentially expressed at 8 WPI, while about 6 genes and 30 lncRNAs were found to be differentially expressed at 20 WPI. In timepoint-based analysis, 25 genes and 73 lncRNAs were found to be differentially expressed in infected 8 verses 20 WPI and 199 genes and 137 lncRNAs were found to be differentially expressed in uninfected 8 verses 20 WPI. Surprisingly, uninfected 8 vs 20 WPI showed highest number of DEGs and DElncRNAs. As per the original paper^4^, this difference is due to the changes in age of the animals during the experiment. The number of genes and lncRNAs differentially expressed at each condition were mentioned in Table 2.

**Table 2:** Table showing the number of differentially expressed genes (DEGs) and differentially expressed lncRNAs (DElncRNAs) obtained in (I) between infected and uninfected and (II) between different time points.

Condition	Infected vs uninfected		8 vs 20 WPI	
	8 WPI	20 WPI	Infected	Uninfected
DEgenes				
Total	6	6	25	199
Up-regulated	5	5	10	50
Down-regulated	1	1	15	149
DElncRNAs				
Total	87	30	73	137
Up-regulated	66	23	35	79
Down-regulated	21	7	38	58

**Figure 2:**
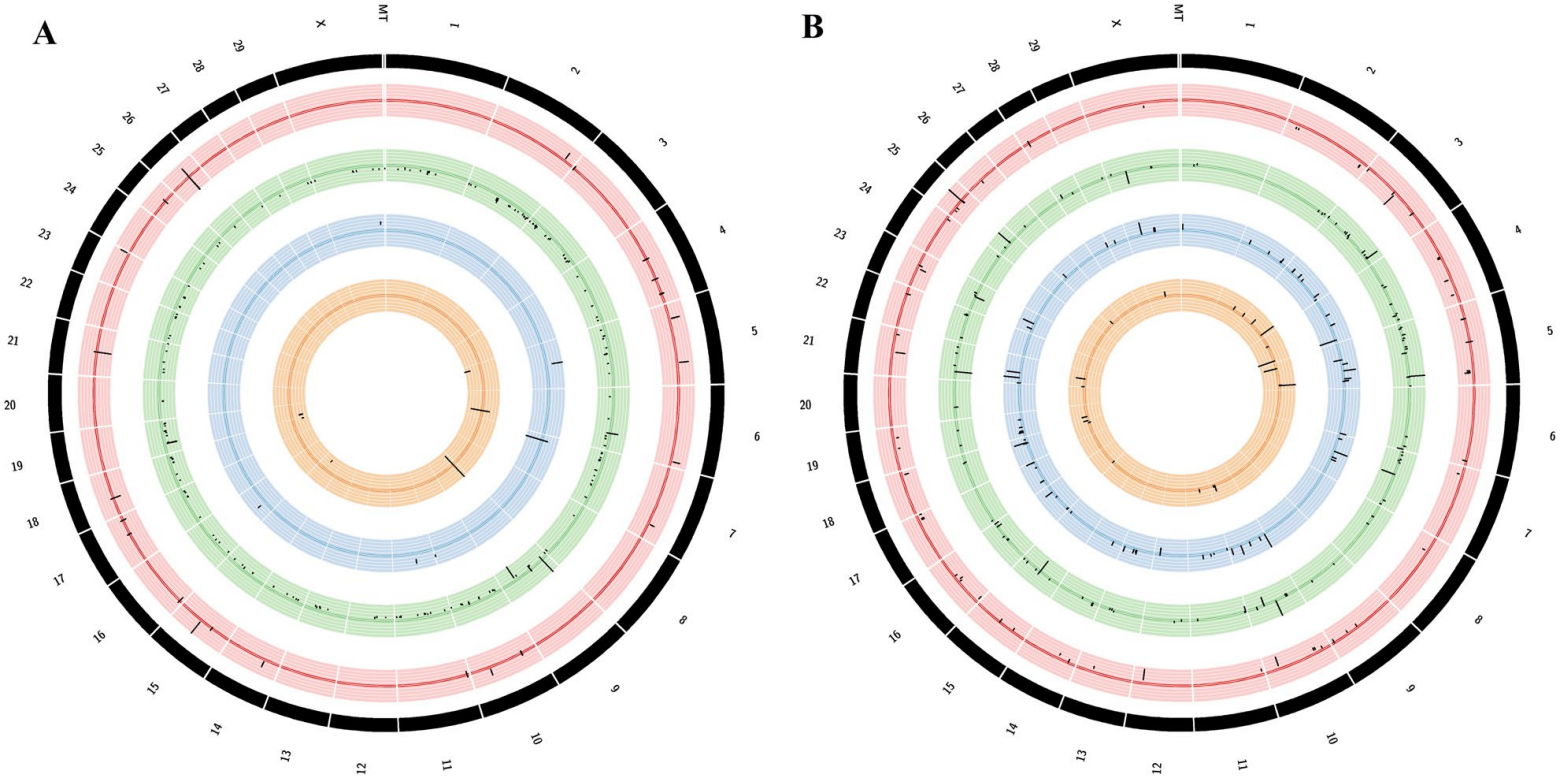
Figure showing the chromosomal localisation of (**A**) differentially expressed genes and (**B**) long noncoding RNAs. The outermost black circle signifies the chromosomes of cow. The inner concentric circles depict the positions of DEGs and DElncRNAs identified in different conditions in the order infected 8 vs 20 WPI, uninfected 8 vs 20 WPI, infected vs uninfected 8 WPI and infected vs uninfected 20 WPI.

The Circos plots, illustrated in Fig. 2A and B, provide a visual representation of the chromosomal localization of differentially expressed genes and lncRNAs under various conditions. In the visualization, the outermost black circle signifies the chromosomes of cow. The inner concentric circles depict the positions of differentially expressed genes and lncRNAs on chromosomes in the order infected 8 vs 20 WPI, uninfected 8 vs 20 WPI, infected vs uninfected 8 WPI and infected vs uninfected 20 WPI. Lines extending outward from the central line signify up-regulation, while lines moving inward denote down-regulation of the genes and lncRNAs. The figure shows that in comparison to genes, more lncRNAs were differentially expressed in two analysis conditions. Time-based analysis showed more differentially expressed genes and lncRNAs, while infected and uninfected based analysis showed fewer genes and lncRNAs. In the infected and uninfected-based analysis, the number of genes remained constant between the two time points, while the count of differentially expressed lncRNAs was higher at 8 WPI compared to 20 WPI. These visualizations provide a clear overview of the genomic distribution and expression patterns of genes and lncRNAs under different conditions and timepoints.

### Gene ontology analysis

**Figure 3:**
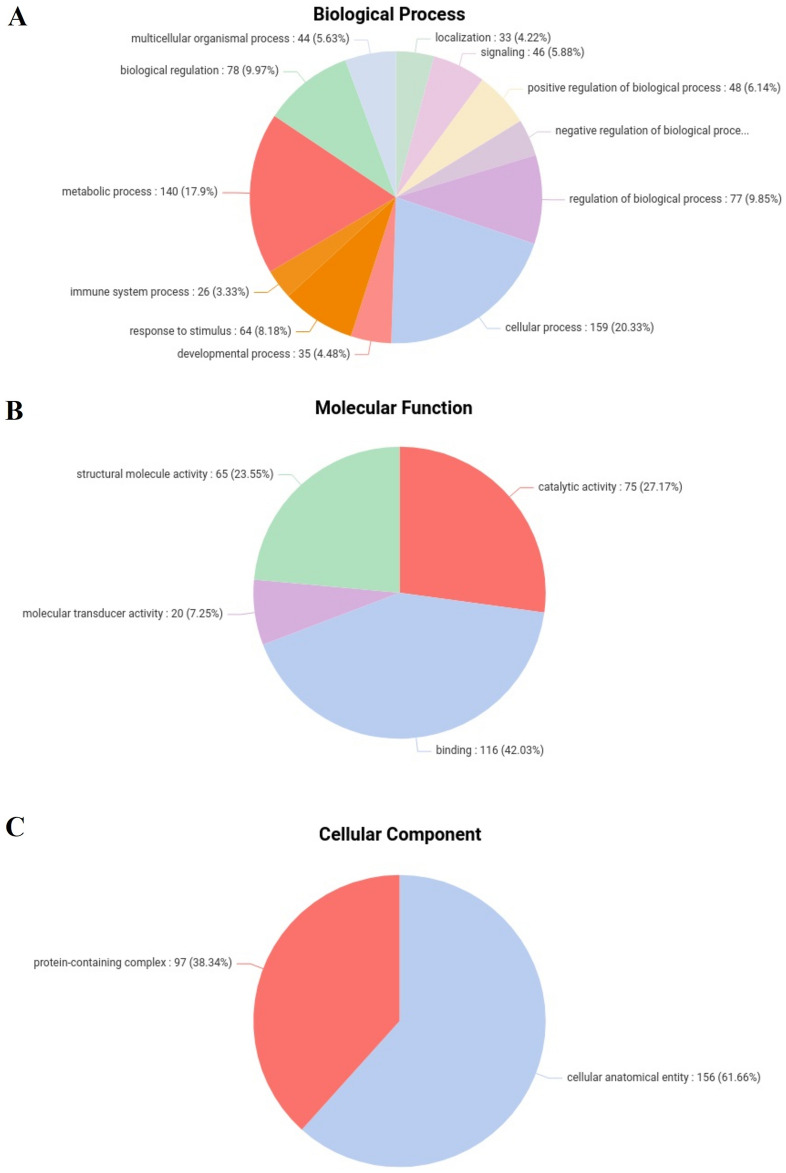
Figure showing the pie charts representing the Level 2 Gene ontologies (GOs) of the differentially expressed genes of all conditions—(**A**) biological process GOs, (**B**) molecular function GOs and (**C**) cellular component GOs.

**Figure 4:**
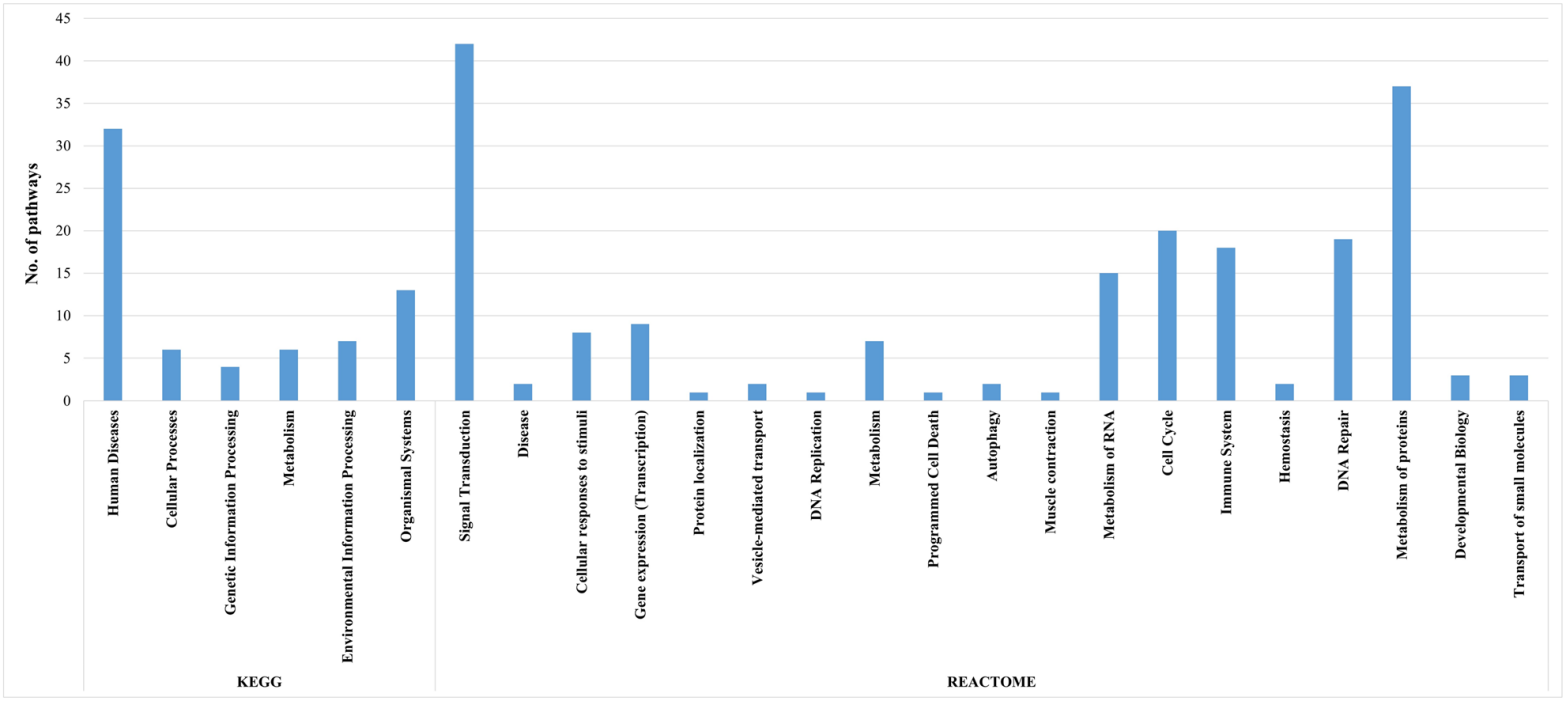
Figure showing the distribution of the categories of pathways annotated using KEGG and Reactome databases.

The gene ontologies were represented in Fig. 3A with level 2 Gene Ontologies (GOs) including biological process, molecular function and cellular component. In the analysis of biological process level 2 GOs, a total of 12 categories were identified. Notably, 20.33% (159) of the genes were associated with cellular processes (GO:0009987), followed by 17.9% (140) annotated with metabolic processes (GO:0008152). Immune system processes (GO:0002376) were represented by 3.2% (25) of the genes. Figure 3B illustrates the annotation of DEGs with 4 distinct molecular function level 2 GOs, with 42.03% (116) of the genes annotated with binding (GO:0005488) and 7.25% (20) with molecular transducer activity (GO:0060089). Figure 3C displays the annotation of DEGs with 2 cellular component level 2 GOs, where 61.66% (156) were associated with cellular anatomical entities (GO:0110165), and 38.34% (97) were linked to protein-containing complexes (GO:0032991). Figure 4 shows the diversity of the pathways of the differentially expressed genes across different categories of the KEGG and Reactome databases. The KEGG database pathway analysis showed that most pathways were in Organismal systems, followed by the environmental information processing category, while very few were in the genetic information processing category. As per the Reactome database, the highest number of pathways were in the signal transduction category, followed by the metabolism of proteins category. In contrast, only one pathway was found in protein localisation, DNA replication, programmed cell death and muscle contraction categories.

Apart from this, a total of 8 different genes were annotated with biological process Immune system process (GO:0002376) and only 1 gene was annotated with pathways in the Reactome immune system category, which were annotated with different biological process GOs including amide biosynthetic process (GO:0043604), peptide biosynthetic process (GO:0043043) and chromatin remodelling (GO:0006338). Supplementary Table 4 offers detailed information on gene ontologies, covering biological process, molecular function, and cellular component, EGGNOG annotation, KEGG/Reactome pathways, and pathway categories of all the differentially expressed genes identified in various conditions. These results enhance our understanding of the functional roles and pathways associated with the differentially expressed genes under diverse conditions.

### Co-expression analysis

In the co-expression analysis using Weighted Gene Correlation Network Analysis (WGCNA), modules were identified under two conditions: (I) between infected and uninfected states and (II) between different time points. No outliers were detected in both infection and timepoint-based analyses. The parameters like soft power and minimum module size were determined based on the data. For certain conditions, when the scale-free topology fit index failed to reach values above 0.8 for reasonable powers, the soft power was selected as 9, following guidance from the WGCNA tutorial and detailed in Supplementary Table 5. In infected-uninfected-based analysis, at 8 WPI, three modules were identified with module sizes ranging from 17 to 58. At 20 WPI, four modules were identified with module sizes ranging from 5 to 16. In timepoint-based analysis, four modules were identified, with module sizes ranging from 15 to 48, representing different time points in infected individuals. In infected-uninfected-based analysis, at 8 WPI, five interactions between differentially expressed (DE) genes and lncRNAs were identified with a threshold value of 0.1. At 20 WPI, 30 interactions were identified with a threshold value of 0.01, indicating weak interactions. In the timepoint-based analysis between different time points of infected individuals, 41 interactions between DE genes and lncRNAs were identified with a threshold value of 0.1. These findings provide insights into the co-expression patterns and interactions between DE genes and lncRNAs under different conditions and time points, shedding light on potential regulatory networks associated with the host's response to infection.

### Cis–trans analysis

In the co-expression analysis of lncRNAs and gene pairs, the cis and trans analyses were conducted. In infection-based analysis, at 8 WPI, no cis-acting pairs were identified. At 20 WPI, two cis-acting pairs were found in which lncRNAs were located within the genes. In timepoint-based analysis between different timepoints of infected individuals, a total of 9 cis-acting pairs were identified. Out of these, one pair had the lncRNA downstream of the gene. Four pairs had the lncRNA upstream of the gene. Four pairs had the lncRNA within the gene. These findings, detailed in Table 3, provide information about the spatial relationships between co-expressing lncRNAs and genes under different conditions and time points.

**Table 3:** Table showing the number of trans, cis and different categories of cis co-expressing interactions between differentially expressed genes and long noncoding RNAs—(I) between infected and uninfected and (II) between different time points.

Condition		Infected vs uninfected		Infected
		8 WPI	20 WPI	8 vs 20 WPI
Total interactions		5	30	41
Trans interactions		5	28	32
Cis interactions	Total	0	2	9
	Downstream	0	0	1
	Upstream	0	0	4
	Within gene	0	2	4
	At 3′ end	0	0	0
	At 5′ end	0	0	0

### Gene–transcription factor interaction analysis

In the analysis of transcription factors, 10 motifs were identified within the differentially expressed genes in both conditions. Three motifs (Motif 1, 2 and 9) with a p-value cut-off of 0.05 were related to the transcription factors. Six different transcription factors were identified to match the three motifs; among them, two belong to the ZNF263 family, and the other four transcription factors were identified as SP2, SP1, NFKB1, and NFATC2. The transcription factors SP2 and SP1 were found to match Motif-1, NFKB1 and NFATC2 were found to be matching to Motif-9 and ZNF263 was found to be matching to both Motif-1 and Motif-2. Supplementary Table 6 provides detailed information about the identified transcription factors, including their associations with specific motifs.

### Gene miRNA analysis

In the miRNA analysis, 125 microRNAs were found to target approximately 48 differentially expressed genes in both analysis conditions. In infected and uninfected based analysis, miRNA bta-mir-326, bta-mir-328, bta-mir-1291, bta-mir-2364, bta-mir-2447, bta-mir-2455, bta-mir-2897, and bta-mir-3431 were found to be targeting one differentially expressed gene at 8 WPI. No miRNAs were found to target differentially expressed genes at 20 WPI in this specific analysis.

### Network visualisation

**Figure 5:**
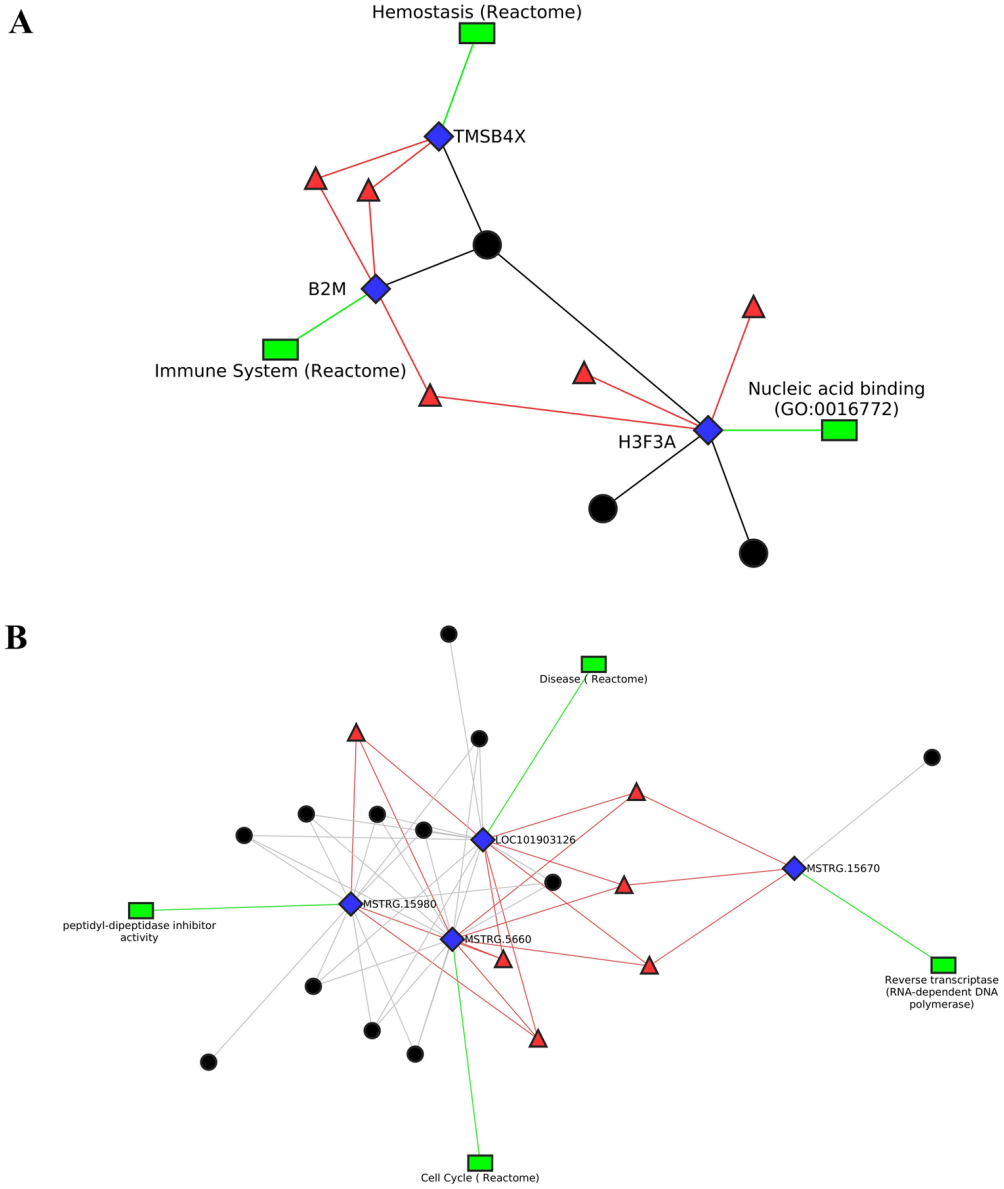
Figure showing the Network diagrams of differentially expressed genes with co-expressing long noncoding RNAs and transcription factors identified in (**A**) between infected and uninfected at 8 WPI and (**B**) between infected and uninfected at 20 WPI.

**Figure 6:**
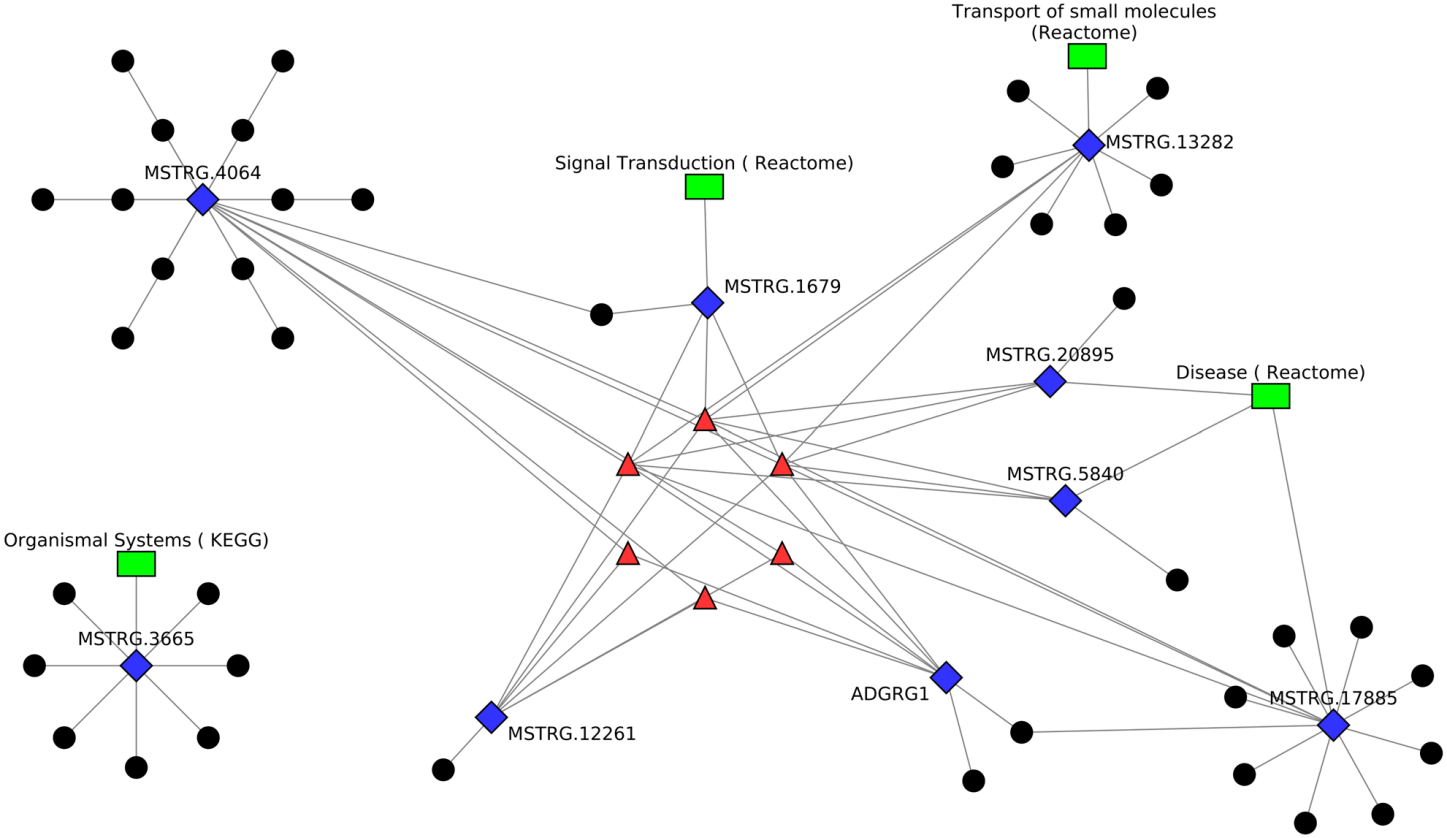
Figure showing the Network diagrams of differentially expressed genes, with co-expressing long noncoding RNAs, Transcription factors identified between different time points of infected (8 vs 20 WPI).

Using the Cytoscape tool with the yfiles organic layout, the interaction network of differentially expressed lncRNAs, co-expressing genes with transcription factors, and associated pathways or biological process GOs was visualized. Figures 5 and 6 depict plots illustrating the comparisons between infected and uninfected conditions and between different time points. In these figures, blue diamond-shaped nodes represent differentially expressed genes, black circular nodes represent differentially expressed lncRNAs, red triangular nodes signify transcription factors interacting with the differentially expressed genes, and green rectangular nodes represent pathways or biological process GOs. In Fig. 5A, represents network obtained data of infected versus uninfected at 8 WPI. This figure shows 3 DEGs, 3 DElncRNAs and 5 TFs. Of the 3 DEGS, gene H3FA was annotated with nucleic acid binding GO and co-expressing with 3 DElncRNAs, gene B2M was annotated with immune system (Reactome) pathway and co-expressing with 1 DElncRNA and gene TMSB4X was annotated with Haemostasis (Reactome) and co-expressing with 1 DElncRNA. In Fig. 5B, network obtained data of infected versus uninfected at 20 WPI. This figure shows 4 DEGs, 12 DElncRNAs and 6 TFs. Of the 4 DEGS, gene Loc101903126 was annotated with Disease (Reactome) and co-expressing with 10 DElncRNAs, gene MSTRG.15670 was annotated with Reverse transcriptase pathway and co-expressing with 1 DElncRNA and gene MSTRG.15980 was annotated with peptidyl-dipeptidase inhibitor activity and co-expressing with 9 DElncRNAs and MSTRG.5660 was annotated with Cell cycle (Reactome) and co-expressing with 8 DElncRNAs. In Fig. 6, network obtained data of infected 8 versus 20 WPI. This figure shows 9 DEGs, 39 DElncRNAs and 6 TFs. Of the 9 DEGS, gene ADGRG1 was unannotated and co-expressing 2 DElncRNAs, gene MSTRG.12261 was unannotated and co-expressing with 1 DElncRNA, gene MSTRG.13282 was annotated with Transport of small molecules (Reactome) and co-expressing with 6 DElncRNAs, gene MSTRG.1679 was annotated with Signal Transduction (Reactome) and co-expressing with 1 DElncRNA, gene MSTRG.17885 was annotated with Disease (Reactome) and co-expressing with 8 DElncRNAs, gene MSTRG.20895 was annotated with Disease (Reactome) and co-expressing with 1 DElncRNA, gene MSTRG.3665 was annotated with Organismal Systems (KEGG) and co-expressing with 7 DElncRNAs, gene MSTRG.4064 was unannotated and co-expressing with 12 DElncRNAs and gene MSTRG.5840 was annotated with Disease (Reactome) and co-expressing with 1 DElncRNA. These visualizations provide a comprehensive overview of the regulatory network, showcasing the relationships between differentially expressed genes, lncRNAs, transcription factors, and associated pathways or biological processes. The yfiles organic layout, based on the force-directed layout paradigm, helps in understanding the structural organization and potential interactions within the complex network. The different shapes and colours of nodes add clarity to the visualization, making it easier to interpret the roles of various elements in the network.

## Discussion

Bovine tuberculosis (BTB) is one of the most significant zoonotic infectious diseases. In this study, the analysis of the peripheral blood transcriptome has provided valuable insights into the regulatory landscape of host response to *Mycobacterium bovis* infection, particularly focusing on the differential expression of genes and lncRNAs during various stages of infection.

In this study, a total of 51,812 long noncoding transcripts were obtained, out of which 21,708 transcripts were found to be matching with the known lncRNAs on the NONCODE database, of which 489 sequences were found to be having 100% coverage and 100% similarity. This analysis identified several unknown and novel long noncoding RNAs. Of these, 46% of the transcripts were found to be intergenic (Class code U), 36% of the transcripts were found to be intronic (Class code I), and 18% were found to be antisense (Class code X). In the analysis of differential expression, a more substantial number of differentially expressed genes and lncRNAs were observed in the timepoint-based analysis, surpassing the findings from the infected vs uninfected analysis. This result suggests a greater variation in transcriptome response between different time points compared to the distinction between infected and uninfected samples. Surprisingly highest number of DEGs and DElncRNAs were identified in uninfected 8 vs 20 WPI, which was reported earlier^4^ to be due to the changes in age of animals during sampling timepoints.

In gene ontology and functional annotation, eight differentially expressed genes were identified with annotations related to immune processes, specifically in the "immune system process (GO:0002376)". Out of these eight immune-related genes, between infected–uninfected based analysis, only one gene, i.e., TMSB4X (Thymosin beta 4 X-linked), was differentially expressed at 8 WPI. Similarly, at 20 WPI, only one gene, i.e., MSTRG.5660, was found. The selected genes were involved in haemostasis (Reactome), cell cycle (Reactome), DNA repair (Reactome) and signal transduction (Reactome) category pathways. Additionally, only two genes were annotated with pathways in the Reactome immune system category. At 8 WPI, only one gene, i.e., B2M (Beta 2 microglobulin), was annotated with immune system (Reactome) pathways and at 20 WPI, only one gene, i.e., EEF1A1 (Eukaryotic translation elongation factor 1 alpha 1), was annotated with immune system (Reactome) pathways.

In timepoint-based analysis, between infected 8 WPI and 20 WPI, six genes, i.e., CD247 (CD247 molecule), CD3E (CD3 epsilon subunit of T-cell receptor complex), MSTRG.3659, MSTRG.9371, MSTRG.9684 and MSTRG.19482 were annotated with immune system GOs, and no genes were annotated with Immune system (Reactome) pathways. These genes were found to be annotated with disease (Reactome), transport of small molecules (Reactome), metabolism of proteins (Reactome), metabolism of RNA (Reactome), cell cycle (Reactome), DNA repair (Reactome) and signal transduction (Reactome). In uninfected 8 vs 20 WPI analysis, genes were found to be annotated with different pathways of a wide range of categories including signal transduction (Reactome), metabolism (Reactome), gene expression (Reactome), DNA repair (Reactome), cell cycle (Reactome), programmed cell death (Reactome), cellular responses to stimuli (Reactome), vesicle-mediated transport (Reactome), protein localization (Reactome), developmental biology (Reactome), DNA replication (Reactome), transport of small molecules (KEGG), cellular processes (KEGG), organismal systems (KEGG), genetic and environmental information processing (KEGG). A few genes were also identified to be annotated with Immune System (Reactome) pathways. This clearly shows the differences between uninfected 8 vs 20 WPI were due to normal changes occurring due to changes in age of the animals.

Apart from immune system related genes, in infection-based analysis at 8 WPI, the genes TMSB4X was annotated with haemostasis (Reactome) and MSTRG.6303 with disease (Reactome) category pathways. At 20 WPI, the genes MSTRG.4066 and LOC101903126 (interferon-induced very large GTPase 1) were annotated with disease (Reactome), and the gene MSTRG.5660 was annotated with cell cycle (Reactome), DNA repair (Reactome) and signal transduction (Reactome). In timepoint based analysis, between infected 8 and 20 WPI, several genes were identified to be annotated with pathways of categories—environmental information processing (KEGG), organismal systems (KEGG), cellular processes (KEGG), metabolism (KEGG), signal transduction (Reactome), transport of small molecules (Reactome), metabolism of proteins (Reactome), metabolism of RNA (Reactome).

The co-expression analysis between DEGs and DElncRNAs showed several co-expression interactions. In infected 8 vs 20 WPI, cis-acting lncRNAs were found to be co-expressing with genes—MSTRG.3665, MSTRG.4064, MSTRG.5840, MSTRG.13282 and MSTRG.17885 which were involved in signal transduction (Reactome), organismal systems (KEGG), disease (Reactome), transport of small molecules (Reactome). Several trans lncRNAs were also identified with same genes. In infection-based analysis at 8 WPI, no cis-acting lncRNAs were identified. Trans lncRNAs were found to be co-expressing with genes—B2M and TMSB4X which were annotated with immune system (Reactome) and haemostasis (Reactome). The gene TMSB4X was also annotated with GO:0002376 (immune system process). At 20 WPI, cis lncRNAs were identified to be co-expressing with gene MSTRG.15980 with no annotation and trans lncRNAs were found to be co-expressing with genes LOC101903126 and MSTRG.5660 which were annotated with disease (Reactome), cell cycle (Reactome), DNA repair (Reactome) and signal transduction (Reactome). The gene MSTRG.5660 was also annotated with GO:0002376 (immune system process). This shows that DElncRNAs which were co-expressing with immune DEGs were identified in infection-based analysis between infected and uninfected samples. While in timepoint-based analysis, no immune DEG was found to be co-expressing with DElncRNAs.

Overall, this study shows the key role of the non-coding part of the genome, which was earlier termed as Junk DNA or Dark matter, in understanding the disease progression, especially in peripheral blood of cattle. The identification of lncRNAs and immune-related genes provides a foundation for further experiments, contributing to the unravelling of regulatory mechanisms in the genetic improvement of cattle. The dynamic changes observed in gene expression over time highlight the importance of considering different stages of infection in future studies. In conclusion, our study contributes significantly to the understanding of host–pathogen interactions in the context of Bovine tuberculosis. Despite the robustness of transcriptomic analysis, several limitations exist in our study. First, the sample size in our study, while sufficient for initial discovery, may limit the generalizability of our findings. Future studies with larger cohorts are essential to validate our results and gain deeper insights into the variability of host responses to BTB. Additionally, our analysis included publicly available data, which, while expanding the scope of our study, introduces variability in experimental conditions and sample characteristics that could impact the robustness of our conclusions. Furthermore, the experimental design, meticulously executed as it was, should be considering potential confounding factors such as age, sex, and genetic background of the cattle studied. Addressing these variables in future research endeavors will be crucial to enhance the reliability and applicability of our findings.

Our study aligns with and extends findings from previous research that emphasizes the pivotal role of lncRNAs in regulating immune responses to bacterial infections. Specifically, our identification of differentially expressed lncRNAs associated with immune pathways corroborates and expands upon existing knowledge in the field of tuberculosis research. However, further comparative analyses with existing datasets and studies are needed to fully contextualize our findings within the broader landscape of host–pathogen interactions. This study provides a basis for future research and potential applications in genetic improvement strategies for cattle and for development of blood-based diagnostic kits. Further validation studies are required for BTB detection in blood.

### Supplementary Information

Supplementary Table 1.

Supplementary Table 2.

Supplementary Table 3.

Supplementary Table 4.

Supplementary Table 5.

Supplementary Table 6.

## Data Availability

Publicly available datasets were analysed in this study. This data can be found here: EBI-ENA database with project ID PRJNA791899. The pipeline used in the study—FHSpipe can be found here: https://github.com/Venky2804/FHSpipe.
